# Do girls with anorexia nervosa have elevated autistic traits?

**DOI:** 10.1186/2040-2392-4-24

**Published:** 2013-07-31

**Authors:** Simon Baron-Cohen, Tony Jaffa, Sarah Davies, Bonnie Auyeung, Carrie Allison, Sally Wheelwright

**Affiliations:** 1 Autism Research Centre, Department of Psychiatry, Cambridge University, 18B Trumpington Rd, Cambridge CB2 8AH, UK; 2 CLASS Clinic, Cambridgeshire and Peterborough NHS Foundation Trust, Cambridge, UK; 3 Phoenix Centre, Ida Darwin, Fulbourn, Cambridge, Cambridgeshire and Peterborough NHS Foundation Trust, Cambridge, UK

**Keywords:** Autistic traits, Anorexia, Autism spectrum conditions, Broader autism phenotype

## Abstract

**Background:**

Patients with anorexia may have elevated autistic traits. In this study, we tested test whether patients with anorexia nervosa (anorexia) have an elevated score on a dimensional measure of autistic traits, the Autism Spectrum Quotient (AQ), as well as on trait measures relevant to the autism spectrum: the Empathy Quotient (EQ), and the Systemizing Quotient (SQ).

**Methods:**

Two groups were tested: (1) female adolescents with anorexia: n = 66, aged 12 to 18 years; and (2) female adolescents without anorexia: n =1,609, aged 12 to 18 years. Both groups were tested using the AQ, EQ, and SQ, via the parent-report adolescent versions for patients aged 12 to 15 years old, and the self-report adult versions for patients aged over 16 years.

**Results:**

As predicted, the patients with anorexia had a higher AQ and SQ. Their EQ score was reduced, but only for the parent-report version in the younger age group. Using EQ-SQ scores to calculate ‘cognitive types’, patients with anorexia were more likely to show the Type S profile (systemizing (S) better than empathy (E)), compared with typical females.

**Conclusions:**

Females with anorexia have elevated autistic traits. Clinicians should consider if a focus on autistic traits might be helpful in the assessment and treatment of anorexia. Future research needs to establish if these results reflect traits or states associated with anorexia.

## Background

Anorexia nervosa (henceforth referred to as ‘anorexia’) is an eating disorder, diagnosed by a refusal to maintain a minimum body weight (>15% below expected body weight), and a preoccupation with food and weight [1]. Anorexia reflects a mix of environmental (social) pressures [2-4], familial dynamics [5], body dysmorphia [6], and genetic predisposition [7,8]. Anorexia largely affects females, has an adolescent onset [9] and is associated with above-average Intelligence Quotient (IQ) [10]. In the present study, we investigated whether there is any link between anorexia and autistic traits, despite the fact that superficially these seem very different. Autism spectrum conditions (ASC) are diagnosed far more often in males than in females [11]. They are neurodevelopmental conditions, diagnosed on the basis of social and communication difficulties alongside unusually narrow interests (pejoratively called ‘obsessions’), strongly repetitive behavior, and a resistance to change [1]. Classic autism is usually diagnosed in early childhood, whereas Asperger syndrome (AS), an ASC in which language development proceeds on time and IQ is at least in the average range, is often diagnosed only later. The recent DSM-5 criteria subsume both of these subgroups under a single heading of ‘autism spectrum disorder’. The notion of the autistic spectrum has been extended from clinical samples to the general population, and it is now recognized that there are individual differences in the number of ‘autistic traits’ right across the population [12,13].

There are several reasons for considering that anorexia and autistic traits may be linked. First, anorexia involves rigid attitudes and behavior, which can be seen as resembling the unusually narrow interests and rigid and repetitive behavior in autism, but in anorexia happen to focus on food or weight. Second, patients with anorexia are often extremely self-preoccupied (about their own weight, or their right to do what they want), and the word ‘autism’ literally means an exclusive focus on the ‘self’. A preoccupation with the self can present as a failure to empathize, for example, with the stress their behavior causes their family, and this resembles the social difficulties in autism. Third, both autism and anorexia show social anhedonia [14,15], deficits in ‘emotional intelligence’ [16,17], difficulties on ‘advanced’ theory of mind tests (such as the ‘Reading the Mind in the Eyes’ test) [18-25], and alexithymia (difficulty in reflecting on one’s own emotion). Fourth, both autism and anorexia involve rigidity on tests of set-shifting [26-30], and excellent performance on tests of attention to detail, such as the Embedded Figures test [31-34]. Finally, both autism and anorexia show atypical structure and function in ‘social brain’ regions, including in the superior temporal sulcus, fusiform face area, amygdala, and orbitofrontal cortex [35].

For these reasons, it is possible that autism and anorexia share common underlying cognitive and neural phenotypes [35,36]. The fact that these two conditions can be comorbid [29] is at least in line with this view, although comorbidity alone does not help us understand why they are associated. The fact that many children with autism are ‘picky eaters’ and resist eating new foods [37] provides another impetus for investigating the link between anorexia and autism further, as does the finding that girls with AS score significantly higher than controls on the Eating Attitudes Test (EAT)-26 [38], a screening instrument for eating disorders [39].

One way to test if autistic traits are elevated in anorexia is to use the E-S framework [42]. This holds that individuals on the autistic spectrum have below-average empathy (E) alongside intact or even superior ‘systemizing’. Empathy is defined as the drive to identify another person’s thoughts and feelings, and to respond to that person’s mental states with an appropriate emotion [40,41]. Systemizing is defined as the drive to analyze or build a system (be it mechanical, abstract, natural, taxonomic, or any other kind of system). Systems are lawful (they follow rules), thus when a person systemizes, they attempt to identify the rules of the system in order to understand it and predict how it will work [41].

Measuring empathy and systemizing has been simplified through the use of a pair of dimensional questionnaires, the Empathy Quotient (EQ) [42] and the Systemizing Quotient (SQ) [43]. These instruments very clearly distinguish people with ASCs from controls, with people with ASC on average scoring significantly lower on the EQ, and people with ASC on average scoring significantly higher on the SQ [44]. The EQ and SQ have been found to predict the number of autistic traits an individual shows, using the Autism Spectrum Quotient (AQ) [44]. The AQ has five subscales (social, communication, attention to detail, attention-switching, imagination) and is a 50-point scale; 80% of people with ASC score above 32 on the AQ [13]. Results from all three of these questionnaires have been independently replicated [45-49].

The EQ and SQ can also be used to calculate an individual’s ‘cognitive type’, as the discrepancy between these two measures can vary in five ways: if EQ is greater than SQ, the individual is said to have a cognitive style of Type E; if SQ is greater than EQ, the person is Type S; if EQ is not significantly different to SQ, the person is Type B (for ‘balanced’); and finally, an individual with more marked discrepancies could be classified as either Extreme Type E or Extreme Type S. Using this taxonomy, more females in the general population score as Type E, more males as Type S, and more people with ASC as Extreme Type S [44,50].The brain basis of these ‘cognitive types’ (a psychometric profile) is beginning to be established [51,52].

One previous study has used the EQ, SQ, and AQ in anorexia [53], testing 22 females with anorexia and 45 female controls. The study found that people with anorexia had a higher number of autistic traits on the AQ (mean ± SD 23.2 ± 7.3) compared with controls (15.3 ± 5.3) (*P*<0.001). The differences appeared most clearly on four of the five subscales. In that study, patients with anorexia did not differ from controls on the EQ and SQ. Because the experimental sample size was relatively small (n = 22), it is important to retest these results, as the earlier study may have been statistically underpowered to reveal any possible differences. In the current study, we tested a sample three times larger (n = 66 females with anorexia).

## Methods

### Participants

Two groups of female adolescents were tested.

1. Adolescents with anorexia: this group comprised 66 female patients with anorexia. The patients’ age range was 12 to 18 years old. They were recruited via seven specialist child and adolescent eating-disorder clinics in the UK. One participant had a diagnosis on the autism spectrum, and so was excluded. All participants had a diagnosis of anorexia according to DSM-IV criteria, as confirmed by their clinician. All clinicians making the diagnosis of anorexia nervosa were specialist psychiatrists in clinics for eating disorders. No information was available on malnutrition status, but instead we collected information on percentage-expected body mass index (BMI), calculated from self-reported age, weight, and height. Because self-reported weight data can be unreliable, BMI was not analyzed as a variable of interest, but the data confirmed that all patients with anorexia had a BMI significantly below the average range. Patients (if over 16 years old) or their parents (if patients were under 16 years old) were asked a set of diagnostic questions in writing. These questions checked whether the patient had been diagnosed with any of the following: anorexia, bulimia, atypical eating disorder, obsessive compulsive disorder, depression, bipolar disorder, attention deficit disorder/attention deficit disorder with hyperactivity, or an ASC. The purpose of this was simply to have converging information from the parent/patient about diagnosis, but the inclusion criterion for the anorexia group was a diagnosis of anorexia nervosa by a specialist clinician.

2. Adolescents without anorexia: this group comprised 1,609 typical female teenagers aged 12 to 18 years old , who were recruited from a general population epidemiological study [54], originally via Cambridgeshire primary schools, or who had provided these data as part of an online study [44]. Screening of this group was via a brief parent-report questionnaire asking if their child had been referred for or been diagnosed with any psychiatric or neurodevelopmental condition.

The age (mean ± SD) of the group with anorexia was 17.85 ± 0.39 years, and that of the group without anorexia was 18.56 ± 3.99. The group with anorexia was significantly younger than the group without anorexia (t = 4.59, *P*<0.05 (equal variances not assumed)). This mean age difference between the groups is less than 1 year, and its significance arises from the larger variance in the control group. Although age has not been found to affect the principal measures of interest (AQ, EQ, or SQ; see below) [55], we entered age as a covariate in all analyses.

### Ethical approval

All participants gave signed consent on their own behalf if they were over 18 years old, and via their parent if they were under 18 years old. Ethics approval was obtained from the Psychology Research Ethics Committee, University of Cambridge.

### Questionnaires

We used the adult and adolescent versions of the AQ [13,56], the EQ, and the Systemizing Quotient-Revised (SQ-R) [44,55]. The mean scores for females in the general population are 15.3 ± 5.7 on the adolescent version of the AQ; 15.5 ± 5.6 on the adult version AQ; 46.6 ± 13.8 on the EQ-Adolescent; 48.0 ± 11.3 on the EQ-Adult; 21.6 ± 12.1 on the SQ-Adolescent; and 51.7 ± 19.2 on the SQ-Adult. The adult versions were used for participants aged 16 years or older, and the adolescent versions were used for participants younger than 16 years. In addition, we assessed patients using the Eating Disorders Examination Questionnaire (EDEQ), which consists of four subscales (eating concern, shape concern, weight concern, and restraint) to confirm the severity of anorexia [57].

### Predictions

We predicted that, relative to controls, patients with anorexia would score higher on the AQ and SQ, but lower on the EQ.

### Procedure

Clinicians at seven specialist child and adolescent eating-disorder clinics in the UK invited patients aged between 12 and 18 years to take part in the study, and distributed questionnaire packs to those who were interested. The questionnaire packs comprised an envelope for the patients (if the patient was >16 years old) or their parents (if the patient was <16 years old). The envelopes included the relevant questionnaire booklet, a consent form, and a freepost envelope for returning the documents. Participants (if aged >16 years) or their parents (if the participant was aged <16 years) completed the questionnaires in their own time. All participants (irrespective of age) gave informed written consent, and in the case of those under 16 years, their parents were also required to consent.

## Results

Mean AQ, EQ, and SQ scores for the patients and female controls are shown in Table 1, stratified by age group.

**Table 1:** Mean scores on the AQ, EQ, and SQ for patients and controls

**Questionnaire**	**Subjects**	**n**	**Mean ± SD**
AQ	Younger patients	24	21.8 **±** 7.9*
	Younger controls	412	11.8 **±** 6.3
	Older patients	42	21.0 **±** 6.8*
	Older controls	1038	15.5 **±** 5.6
EQ	Younger patients	24	44.7 **±** 16.4*
	Younger controls	571	51.2 **±** 14.3
	Older patients	42	49.6 **±** 9.7
	Older controls	1038	48.0 **±** 11.3
SQ	Younger patients	24	39.7 **±** 14.1*
	Younger controls	571	33.4 **±** 15.2
	Older patients	42	64.9 **±** 24.9*
	Older controls	1038	51.7 **±** 19.2

AQ, Autism Spectrum Quotient; EQ, Empathy Quotient; SQ. Systemizing Quotient. * Significant group difference (*P*<0.05).

### AQ

**Figure 1:**
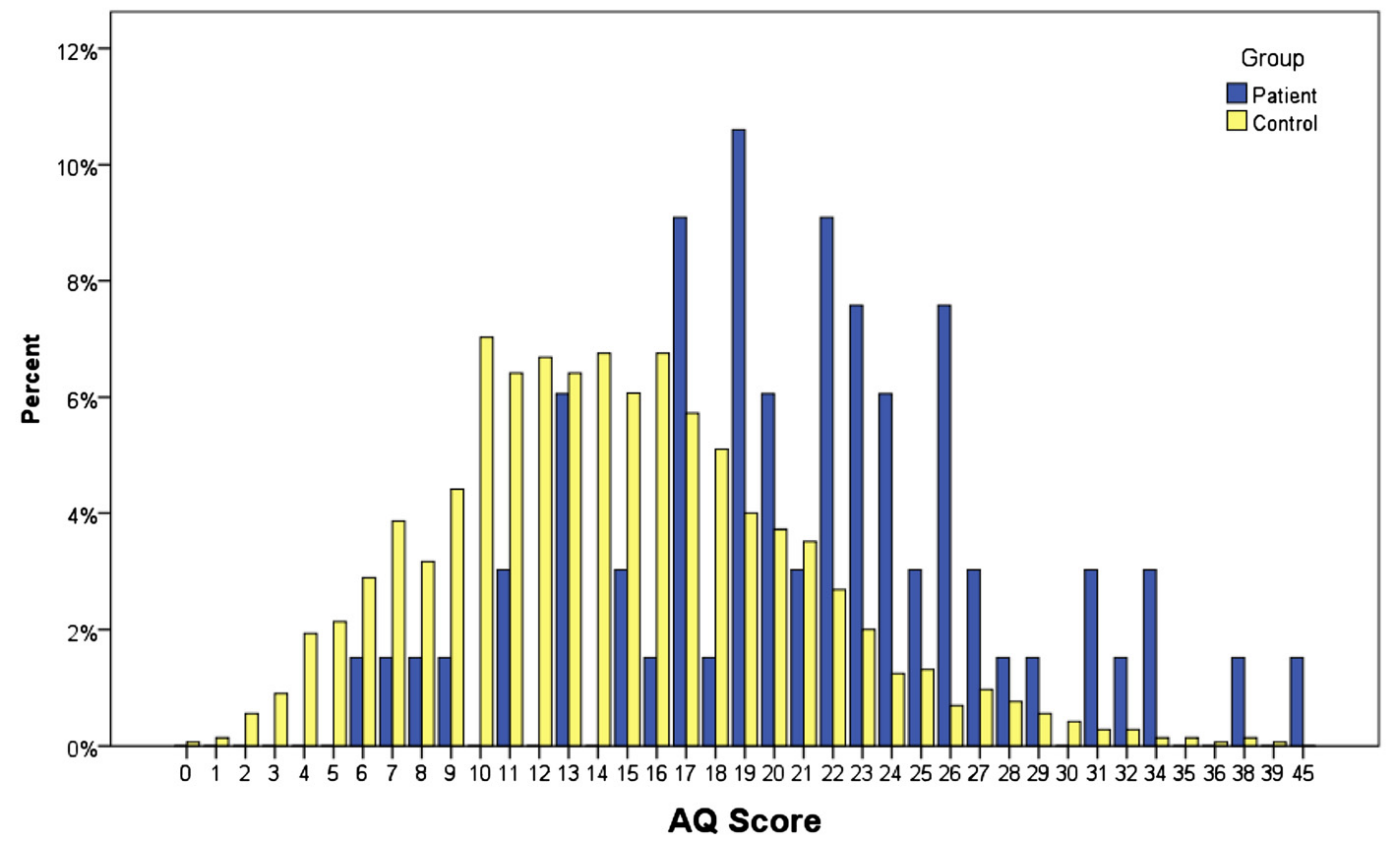
Distribution of Autism Spectrum Quotient (AQ) scores.

The parent-report version of the AQ (used for patients <16 years old) and the self-report version of the AQ (used for patients >16 years old) have the same range (0 to 50), with comparable means and standard deviations [13,56], so the data from both versions of the AQ could be analyzed together. No statistical outliers were seen. As predicted, the mean AQ score for patients was significantly higher than that of the control group, with a large effect size (t = 8.93, *P*<0.001 (equal variances assumed)), Cohen’s d = 1.03). See Figure 1 for the distribution of AQ scores. This group difference remained when the AQ-adolescent and the AQ-adult data were analyzed separately: AQ-Adult (t_(1078)_ = 6.17 (equal variances assumed), *P*<0.001, Cohen’s d = 0.88; and AQ-Adolescent t_(1066)_ = 3.89, *P*<0.001 (equal variances assumed), Cohen’s d = 0.68, respectively).

**Figure 2:**
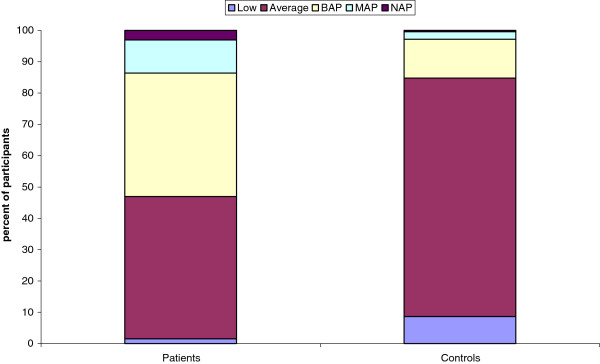
Percentage of participants with each Autism Spectrum Quotient (AQ) phenotype.

Using the AQ, we calculated the percentage of participants defined with what we have previously called the ‘broader autism phenotype’ (BAP: >1 SD above the typical population mean), the ‘medium autism phenotype’ (MAP: >2 SDs above the typical population mean) and the ‘narrow autism phenotype’ (NAP: >3 SDs above the typical population mean), using a method described previously [58]. Low (AQ scores >1 SD below the mean) and average (defined as AQ scores within 1 SD) categories were also calculated. Comparisons of the AQ-defined categories using z-tests for proportions showed that there were significantly larger proportions of controls in the low (z = 2.08, *P*<0.05) and average (z = 5.96, *P*<0.05) categories. There were larger proportions of the patients in the BAP (z = 5.89, *P*<0.05), MAP (z = 3.75, *P*<0.05), and NAP (z = 2.79, *P*<0.05) categories compared with controls (Figure 2, Table 2).

**Table 2:** Proportion of participants in each AQ-defined category

**AQ category**	**Patients**	**Controls**
Low	1.4*	8.6
Average	43.5*	76.1
BAP	37.7*	12.4
MAP	10.1*	2.4
NAP	2.9*	0.4

AQ, Autism Spectrum Quotient; BAP, Broader Autism Phenotype; MAP, Medium Autism Phenotype; NAP, Narrow Autism Phenotype. * Significant group difference (*P*<0.05).

### EQ and SQ

Unlike the AQ, the parent-report and self-report versions of the EQ and SQ cannot be combined because they have a different number of items, produce very different mean scores, and have different ranges. Therefore, in the subsequent analyses, the results are reported for the two age groups separately. A significant difference in EQ scores for the 12 to 15 years age range was seen, with patients scoring lower than controls, giving a medium effect size (t(593) of 2.17 (*P*<0.05 (equal variances assumed), Cohen’s d *=* 0.68). No differences were found between patients and controls on the EQ in the patients aged 16 to 18 years (*P*>0.05).

On the SQ, both patient groups scored higher than controls (12 to 15 years: t_(593)_*=* 2.01, *P*<0.05 (equal variances assumed), with a medium effect size (Cohen’s d *=* 0.44); 16 to 18 years: (t_(1078)_*=* 4.32, *P*<0.001 (equal variances assumed), also with a medium effect size (Cohen’s d *=* 0.59)).

In addition, the percent of participants showing each ‘cognitive type’ was calculated by first computing standardized (normalized) scores on the EQ and SQ, and then using differences between standardized scores to calculate the five ‘cognitive types’ used previously (Types B, E, S, Extreme E or Extreme S) [44,55], summarized as follows:

$$\begin{array}{lll} \text{Standardized}\;E & = & \left(\left(\text{observed}\;\mathrm{EQ}‒\text{mean}\;\mathrm{EQ}\;\text{for}\;\text{typical}\;\right)\right)\\ & & \;\left(\text{population}\right)/\text{maximum}\;\text{attainable}\;\\ & & \;\left(\text{score}\;\text{for}\;\mathrm{EQ}\right) \end{array}$$

$$\begin{array}{c} \text{Standardized}\;S=\left(\left(\text{observed}\;\mathrm{SQ}‒\text{mean}\;\mathrm{SQ}\;\text{for}\;\text{typical}\;\right)\right)\\ \;\left(\text{population}\right)/\text{maximum}\;\text{attainable}\;\\ \;\left(\text{score}\;\text{for}\;\mathrm{SQ}\right) \end{array}$$

The standardized E and S variables were used to produce a difference score (D). This new variable was defined as follows:

$$D=\left(S-E\right)/2,$$

where D is the difference between the normalized SQ and EQ scores.

The proportion of participants with each brain type is shown in Table 3. Comparisons using z-tests between proportions of patients and controls showing each cognitive type showed no differences in the Extreme E, Type E, Type B, or Extreme S categories. The only significant difference was found in Type S, with a larger proportion of patients exhibiting Type S compared with controls (z *=* 2.26, *P*<0.05 and z *=* 1.77, *P*<0.05, respectively).

**Table 3:** Proportion of participants with each cognitive brain type

**Brain type**	**12 to 15 years old**		**16 to 18 year-old patients**	
	**Patients**	**Controls**	**Patients**	**Controls**
Extreme E	0	3.0	2.4	4.3
Type E	33.3	40.1	38.1	44.8
Type B	16.7	31.9	23.8	29.3
Type S	45.8*	23.5	33.3*	20.7
Extreme S	4.2	1.6	2.4	0.9

* Significant group difference (*P*<0.05).

### EDEQ

On the EDEQ, a one-sample *t*-test showed that patients with anorexia scored significantly higher on all subscales than published norms [59] for Restraint (t *=* 6.81, *P*<0.001), Eating Concern (t *=* 7.93, *P*<.001), Shape Concern (t *=* 15.39, *P*<0.001), Weight Concern (t *=* 5.70, *P*<0.001), and Global (t *=* 9.32, *P*<0.001) tests (Table 4). There were no correlations between EDEQ subscales and the AQ, EQ, or SQ (all *P*>0.05).

**Table 4:** **Eating Disorders Examination Questionnaire** (**EDEQ) scores**

**Subscale**	**Mean ± SD**	
	**Patients**	**Controls**
Restraint	3.4 ± 1.8*	1.6 ± 1.2
Eating concern	3.2 ± 1.6*	1.3 ± 1.3
Shape concern	4.5 ± 1.7*	0.6 ± 0.9
Weight concern	3.8 ± 1.9*	2.2 ± 1.6
Global	3.8 ± 1.6*	1.6 ± 1.4

Norms from Carter et al. [59]. *Significant group difference *P*<0.01).

## Discussion

This study set out to extend a previous study [53] that examined whether patients with anorexia nervosa have an elevated number of autistic traits, as measured on the AQ. In addition, it aimed to test whether these patients also have an atypical cognitive profile, as reflected by a discrepancy between their empathy (measured using the EQ) and their systemizing (measured using the SQ). We confirmed the earlier finding that AQ is indeed elevated in patients with anorexia. Patients with anorexia were also more likely to show the broader (BAP) and medium (MAP) autism phenotypes [58]. In addition, patients with anorexia had a significantly higher SQ and a significantly lower EQ (in the younger sample only); the reason these may not have been significant in the previous study is likely to be due to power, with our study being three times larger than the previous study.

In terms of ‘cognitive types’, patients with anorexia were significantly more likely to be Type S (systemizing significantly better than empathy). Because Type S is usually more common in males [44,55], the finding that females with anorexia show this more ‘masculinized’ cognitive profile is anomalous, and warrants further exploration. The link between Type S and anorexia merits some consideration. A person who is ‘obsessed’ with calories, body size, and body weight may be directing their strong systemizing towards the domain of food or food intake, and indeed may have systemized the relationship between food and weight to a high degree of detail. One view is that their anorexia is then the result of their systemizing having latched onto this particular domain, the implication being that if their systemizing had latched onto a different domain, their behavior would not necessarily have resulted in anorexia, but instead would have had a different obsessive focus. Type S could represent a vulnerability factor for AS in girls. The Type S profile could also reflect below-average empathy, which in the case of anorexia may be manifested by an above-average preoccupation with the self (body shape, weight, appearance) rather than the thoughts and feelings of others.

An implication of the present study is that treatment should focus on the cognitive profile of patients, not just their behavior. Doubtless, most clinicians already consider cognitive factors, but this study draws attention to specific cognitive factors (systemizing, empathy, and autistic traits). The present results highlight that while their AQ score is well below the levels seen in diagnosed autism or AS, people with anorexia on average have some social and empathy difficulties, alongside some narrow interests and resistance to change. Clinicians may find it useful to shift the focus of intervention away from the battle over weight and towards helping the patient to recognize that they have a mind that is more attracted to systems and less to people’s emotional lives. Recognizing what information is easier to process (systematic information) and what is harder to process (other people’s emotions) may be helpful in developing useful coping strategies. This is in line with the current trends or thinking in the neuropsychology of anorexia, and the application of cognitive remediation therapy that targets cognitive style [60].

Limitations of the current study include the following.

1) We cannot report much detail about diagnosis (for example,, duration of anorexia) because this information was not collected.

2) We cannot report a Table of mean weight and height for both groups, as this was unavailable for some participants.

3) The current samples were self-selecting, so it will also be important to test these findings in samples of patients who may be more representative.

4) This study relied on questionnaires, and it will be important to validate these findings using performance measures.

5) We could not measure IQ, so its role, if any, is unknown.

6) We cannot exclude the possibility that some of the control adolescents might have had eating and weight issues even though their EDEQ group average score was significantly lower than that of the patient group. However, if this were the case it would have had the effect of reducing group differences.

7) Because of the wide age range of participants, we necessarily had to use different versions of the questionnaires, which means the measures were more heterogeneous than would have been the case if we had studied a narrower age range.

8) We cannot tell if the present results reflect the consequences of starvation, which is known to make the individual more self-focused and socially withdrawn. It will be important in future to test if a similar profile on these instruments is seen even in ‘recovered’ patients with anorexia. This may elucidate whether these profiles are more related to ‘state’ or ‘trait’. Equally, patients with anorexia are frequently amenorrheic, so future research could explore the role that hormonal status plays in these psychometric profiles.

9) The absence of a clinical control group means that we do not know if the findings from the anorexia group are specific to that condition.

## Conclusion

In conclusion, while clinicians must continue to focus on low weight in patients with anorexia because this can be life-threatening, an understanding that some patients may have a different cognitive style, one prone to an obsessive focus on systems and a self-focused turning away from other people, may open up new avenues for both treatment and etiological research for anorexia.

## Abbreviations

AQ: Autism Spectrum Quotient; BMI: Body mass index; ASC: Autism spectrum conditions; AS: Asperger syndrome; DSM: Diagnostic and Statistical Manual of Mental Disorders; EAT: Eating Attitudes Test; EQ: Empathy Quotient; MAP: Medium Autism Phenotype; NAP: Narrow Autism Phenotype; SQ: Systemizing Quotient.

## Competing interests

The authors declare they have no competing interests.

## Authors’ contributions

SBC and TJ designed the study; SD and SW coordinated data collection; and CA and BA helped with data analysis. All authors contributed to the drafting of the manuscript.
